# Effects of heat-killed *Bifidobacterium longum* KABP-042 and *Lactiplantibacillus plantarum* KABP-051 on cognitive performance in typically developing school-aged children: a placebo-controlled, randomized double-blind parallel-group trial

**DOI:** 10.1186/s40795-026-01446-6

**Published:** 2026-08-05

**Authors:** Naoki Arai, Shinichi Honda, Yuji Tominaga, Jinko Sawashita, Kyoko Tanaka, Isao Nagaoka, Takahiro Ueda

**Affiliations:** 1 Supplement & Probiotics Research Laboratories, Kaneka Corporation, 1-8, Miyamae-cho, Takasago-cho, Takasago, Hyogo 676-8688 Japan; 2 Pharma & Supplemental Nutrition Solutions Vehicle, Kaneka Corporation, Tokyo, Osaka Japan; 3 Department of Pediatrics, Juntendo University Faculty of Medicine, Tokyo, Japan; 4 Graduate School of Medical Science, Juntendo University, Chiba, Japan

**Keywords:** Postbiotics, cognitive function, Gut-brain axis, BDNF, Child’s brain, *Bifidobacterium longum*, *Lactiplantibacillus plantarum*

## Abstract

**Background:**

Increasing evidence suggests that the gut-brain axis plays a critical role in cognitive and behavioral development. However, clinical evidence supporting cognitive benefit of probiotics and postbiotics in typically developing children remains limited. This study evaluated the effects of a postbiotic formulation containing heat-killed *Bifidobacterium longum* KABP-042 and *Lactiplantibacillus plantarum* KABP-051 on brain-related outcomes in school-aged children.

**Methods:**

This study randomly allocated 60 typically developing children (aged 8–10 years) to receive either a daily jelly containing heat-killed KABP-042 and KABP-051 (5 × 10^9^ cells each) or a placebo for 12 weeks. The primary endpoints were changes in working memory (digit span), inhibitory control (Go/No-go task), and cognitive flexibility measured by the Stroop test. Secondary endpoints included autonomic nervous system (ANS) activity, emotional and behavioral assessment, and gut microbiota composition.

**Results:**

Participants who received the postbiotic intervention demonstrated significantly higher accuracy (*p* = 0.019) and lower error rates (*p* = 0.004) in the incongruent condition of the Stroop test at 12 weeks compared to placebo, while reaction time was unchanged. No significant between-group differences were observed in working memory, inhibitory control, ANS activity, emotional and behavioral outcomes, and gut microbiota composition, although changes in beta diversity were significantly greater in the placebo group (*p* = 0.049).

**Conclusions:**

Twelve weeks of postbiotic intervention was associated with enhanced accuracy-related Stroop task performance, particularly in the incongruent condition, in typically developing children, whereas other cognitive outcomes were unaffected. These preliminary findings suggest that supplementation with KABP-042 and KABP-051 may influence selected aspects of cognitive task performance in children. Further investigation into the underlying mechanisms and long-term outcomes is warranted.

**Trial registration:**

This study was registered with the University Hospital Medical Information Network Clinical Trials Registry (UMIN-CTR; ID: UMIN000058366).

**Supplementary Information:**

The online version contains supplementary material available at https://doi.org/10.1186/s40795-026-01446-6.

## Introduction

Based on the interaction between gastrointestinal microbiota and neurological function, widely recognized as the gut-brain axis, an increasing number of studies have attempted to influence brain function via the consumption of lactic acid bacteria [1, 2]. A variety of intervention trials using probiotics have demonstrated their efficacy and reported an improvement in brain function, particularly memory and cognitive decline [3–5], as well as alleviation of mental symptoms such as depression [6, 7]. In addition to probiotic preparations, the beneficial health functions of inactivated bacteria, also called postbiotics, have been well investigated [8, 9]. Postbiotic preparations contain various bioactive components, including structural constituents of bacterial cells and metabolites [10], and have attracted increasing attention, particularly in the field of dietary supplements, owing to their advantages in terms of safety and practical use. Collectively, these findings suggest that the gut-mediated modulation of brain function represents a promising strategy for cognitive improvement.

Accordingly, various molecules involved in gut–brain interactions, including neurotransmitters such as serotonin and γ-aminobutyric acid, short-chain fatty acids, immune-related cytokines, and microbial metabolites, have been investigated [11]. Among the factors potentially influenced by gut-derived signals, brain-derived neurotrophic factor (BDNF) has attracted attention because of its role in neuroplasticity and cognitive function [12, 13]. In our previous preclinical study, oral administration of heat-killed lactic acid bacterial strains, *Bifidobacterium longum* KABP-042 and *Lactiplantibacillus plantarum* KABP-051 restored brain BDNF production and ameliorated memory impairment [14]. Although these findings suggest a potential involvement of gut-brain signaling pathways, the relevance of these observations to cognitive function in humans remains unclear.

Growing evidence supports the effect of microbiome-targeted interventions on brain function in adults, however, clinical evidence in typically developing children remains limited. Childhood is a critical period of brain development and maturation [15, 16], and the establishment of safe, evidence-based nutritional approaches to support cognitive function in this population is of considerable interest. In this study, we aimed to examine the effects of heat-killed KABP-042 and KABP-051 on cognitive function in typically developing school-aged children, through an intervention trial. We also aimed to explore the effects of these postbiotics on emotional and behavioral outcomes and to examine potential associations between the gut and brain through autonomic nervous system measurements and gut microbiota analysis.

## Methods

### Study design and participants

**Fig. 1:**
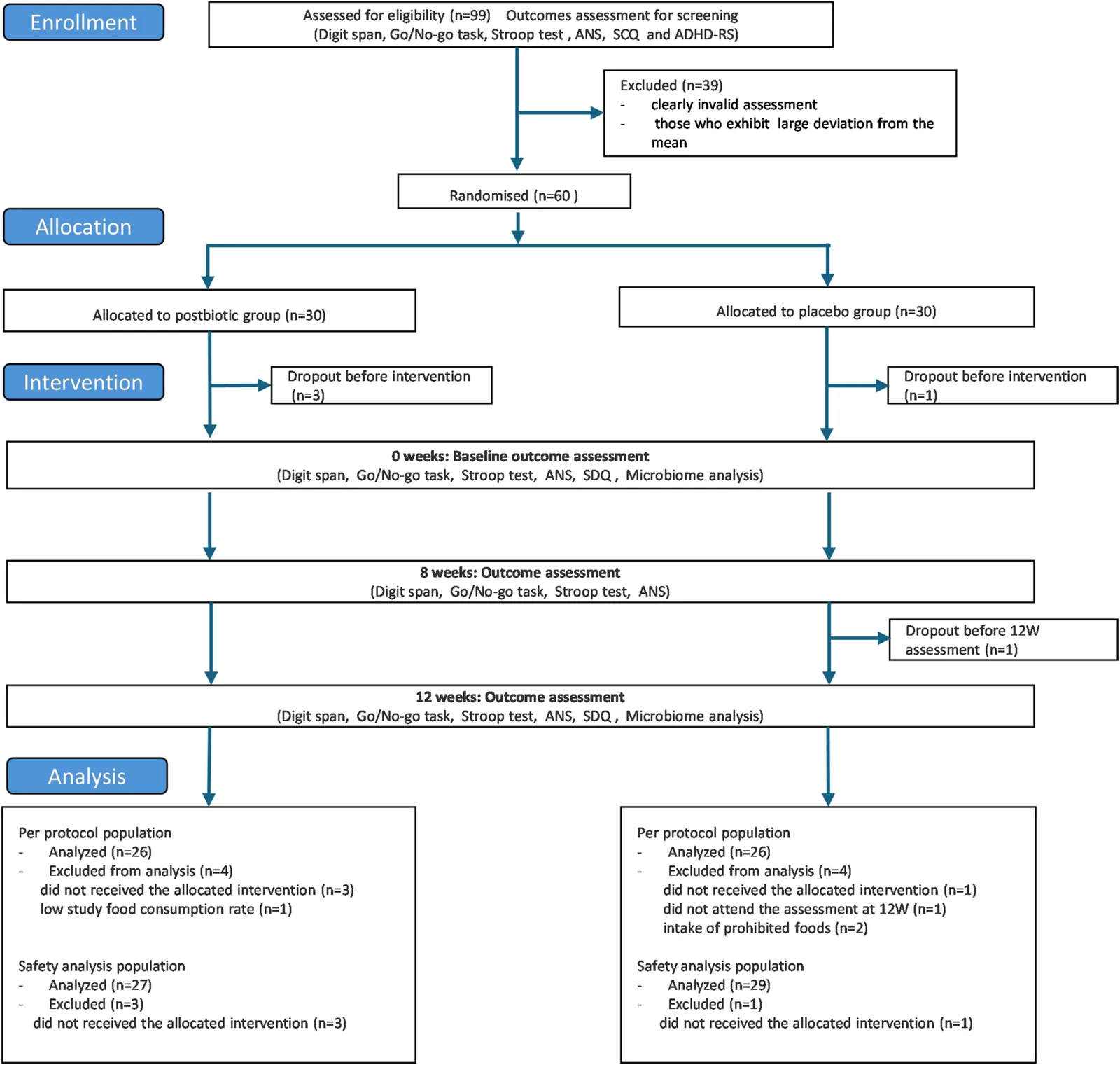
Flow diagram of participants through the phases of a parallel randomized trial comparing postbiotic and placebo groups

This prospective, double-blind, randomized, placebo-controlled study involved two parallel groups and was performed from September 2025 to December 2025 under the control of a contract research organization (Mibyou Life Science, Inc., Tokyo, Japan) commissioned by the Kaneka Corporation (Osaka, Japan). This clinical study was performed in Tokyo, Japan, in accordance with the Declaration of Helsinki and the Ethical Guidelines for Medical and Health Research Involving Human Subjects. The study protocol was approved by the Mibyou Research Ethics Review Committee (June 6, 2025; approval no. 160000010). The study was registered in the University Hospital Medical Information Network Clinical Trials Registry ( ID: UMIN000058366; date of registration: April 1, 2026). Figure 1 shows a flow diagram of the participants in this study.

Participants were recruited from registered monitors (parents) of a Japanese market research company (Macromill Inc., Tokyo, Japan) based on the predefined inclusion and exclusion criteria (Table 1). After a complete description and comprehensive explanation of the procedures, the purpose of the study, and the reassurance of confidentiality, written informed consent was obtained from the parents and oral assent was obtained from the children themselves. We recruited typically developing Japanese school-aged children aged between 8 and 10 years (third- and fourth-grade children in elementary school). In this study, “typically developing children” were operationally defined as children who had not been previously diagnosed with autism spectrum disorder (ASD) or attention-deficit/hyperactivity disorder (ADHD), and who showed no clinically relevant traits based on standardized screening questionnaires. A screening survey was conducted after informed consent and assent were obtained. First, information on sex, date of birth, height, body weight, medical history, surgical history, subjective and objective symptoms, allergies, use of medications and dietary supplements, participation in extracurricular learning activities (e.g., lessons), family structure (parents and siblings), number of household members, household income, parental employment status, history of preterm birth, and birth weight were collected. Subsequently, parents were asked to complete the Social Communication Questionnaire (SCQ) [17] and Attention-Deficit/Hyperactivity Disorder Rating Scale-IV (ADHD-RS-IV) to screen out children with potential ASD or ADHD traits [18]. Participants with scores of ≥ 8 on the SCQ were excluded. Participants with inattention subscale scores of ≥ 11 for males or ≥ 7 for females, hyperactivity/ impulsivity subscale scores of ≥ 13 for males or ≥ 9 for females, or total scores of ≥ 22 for males or ≥ 15 for females on the ADHD-RS were excluded. Together, these comprehensive screening criteria helped ensure a relatively homogeneous cohort of typically developing children for the assessment of cognitive outcomes. In total, 99 participants underwent an outcome assessment (measurement of autonomic nervous system (ANS) activity and cognitive function (digit span, Go/No-go task and Stroop test)) for screening. Participants with invalid assessments were excluded. Furthermore, individuals at the upper and lower extremes of the cognitive performance score distribution were excluded, and a predefined sample of 60 participants was selected for enrollment in a subsequent clinical trial.

**Table 1:** Inclusion and exclusion criteria in the present study

Inclusion criteria
1) Individuals who are native Japanese speakers, currently enrolled in regular third- or fourth-grade elementary school classes at the time of consent (born between April 2, 2015 and April 1, 2017)
2) Individuals who are not receiving any medical treatment
3) Individuals who are able to attend scheduled assessments and complete required records
4) Individuals who, along with their legally authorized representatives (parents/guardians), have received sufficient explanation about the study and have voluntarily provided written informed consent

Exclusion criteria
1) Individuals diagnosed with any neurodevelopmental disorders, intellectual disabilities, or other psychiatric conditions
2) Individuals with a history of serious disorders related to glucose metabolism, lipid metabolism, liver function, kidney function, heart, circulatory system, respiratory system, digestive system, endocrine system, immune system, or nervous system
3) Individuals with visual or auditory impairments or related disorders
4) Individuals with allergies to drugs or foods that may affect the study
5) Individuals who are unable to refrain from consuming specified health foods, including Foods for Specified Health Uses, foods with functional claims, or other dietary supplements during the study period
6) Individuals who are unable to consume the test food as instructed
7) Individuals currently participating in another clinical trial or investigational study
8) Individuals who are unable to follow the instructions of the principal investigator or are deemed unsuitable for participation by the principal investigator

### Intervention

The participants were randomly allocated into two groups using a stratified permuted block randomization method. The 60 participants selected by the investigator after screening were stratified by sex, grade, digit span score, Go/No-go task score, and Stroop task score. Within each stratum, participants were randomly assigned to either the postbiotic or placebo group in a 1:1 ratio using permuted blocks. The randomization sequence was generated by an independent researcher who was not involved in participant recruitment or assessment. Allocation was concealed using sequentially numbered, opaque, sealed envelopes, which were opened only after participant allocation. Investigators and participants remained blinded to the assignment until the end of the study. After the outcome assessment at baseline (0 week), participants in the postbiotic group received one sachet per day of a jelly containing heat-killed cells of *B. longum* KABP-042 (5 × 10^9^ cells) and *L. plantarum* KABP-051 (5 × 10^9^ cells) for 12 weeks, whereas those in the placebo group received one sachet per day of a jelly without postbiotics for the same period. Postbiotic and placebo jellies were indistinguishable in terms of appearance and taste. Participants (or their parents) recorded daily intake of the study product in a diary throughout the intervention period. Compliance was assessed based on diary records and by counting returned unused sachets at each study visit.

### Study product preparation and quality control

The postbiotic jelly was manufactured by Sunsho Pharmaceutical Co., Ltd. (Shizuoka, Japan) under commission from Kaneka Corporation (Osaka, Japan). Briefly, the freeze-dried preparations of *B. longum* KABP-042 and *L. plantarum* KABP-051, supplied by AB Biotics S.A. (Sant Cugat del Vallès, Spain), were suspended in water at 5% (w/v) and heat-inactivated at 90℃ for 30 min. Complete inactivation was confirmed by the absence of colony formation on appropriate culture media, and the resulting heat-killed preparations were incorporated into the flavored jelly matrix so that each sachet (10 g) contained 5 × 10^9^ heat-killed cells of KABP-042 and 5 × 10^9^ heat-killed cells of KABP-051. The jelly matrix consisted mainly of sweeteners (sucralose, acesulfame potassium, and xylitol), pH-adjusting agents, thickening polysaccharides, flavoring agents, emulsifiers, and purified water. The placebo jelly was identical in composition, appearance, flavor, and packaging but did not contain bacterial cells. The products were manufactured under quality-controlled conditions, and product quality was periodically monitored by assessing appearance, flavor, hygienic quality, and total bacterial cell counts to confirm product stability.

### Endpoints and outcome assessments

The primary endpoint was the change in cognitive task scores after 8 and 12 weeks of intervention, as assessed using the digit span (working memory), Go/No-go task (response inhibition), and Stroop test (cognitive flexibility). Secondary endpoints included changes in ANS activity, emotion and behavior (the score of Strengths and Difficulties Questionnaire (SDQ)) and gut microbiota composition. Outcome assessments were conducted at baseline, and weeks 8 and 12 for primary outcomes. Among the secondary outcomes, ANS activity was assessed at all three time points, whereas SDQ and gut microbiota analyses were performed at baseline and week 12. Primary outcomes and ANS activity were assessed at the study site, and the parents completed the SDQ on-site. Fecal samples were collected before the visit and directly transferred to an external analytical laboratory.

Efficacy analyses were conducted on the per-protocol population, which included participants who completed the 12-week intervention with adequate compliance and without major protocol violations. Participants with low study food intake or prohibited food consumption, as confirmed by diary records and investigator reviews, were excluded from the per-protocol analysis according to predefined criteria. No exclusions were made based on the outcome assessments. Due to the exploratory nature of this study, an intention-to-treat (ITT) analysis was not performed.

### Digit span

The digit span test, a subtest of the Wechsler Intelligence Scale for Children (WISC) [19], was performed in accordance with the Japanese WISC-V protocol. Participants listened to prerecorded digit sequences presented through headphones at 1-s intervals and verbally recalled the sequence after an auditory response cue. The test consisted of forward and backward conditions. In the forward test, which assesses short-term memory, participants recalled the digits in the same order as presented. In the backward test, which assesses working memory, participants recalled the digits in reverse order after completing two practice trials. Testing began with a two-digit sequence, with two trials administered for each digit length. The sequence length increased until both trials at a given length were answered incorrectly. Forward and backward scores were calculated as the total number of correctly recalled trials.

### Go/No-go task

Response inhibition was assessed using a computerized Go/No-go task implemented with the Multi-Trigger System (MTS0411V; Medical Try System Co., Ltd., Japan) based on established paradigms widely used in Japanese pediatric populations [20, 21]. The task consisted of three phases: acquisition, discrimination, and reverse-discrimination. In the acquisition phase, participants learned the initial response rule by responding to a blue stimulus. In the discrimination phase, participants responded only to blue stimuli (Go) and withheld responses to red stimuli (No-go). In the reverse-discrimination phase, the stimulus-response contingency was reversed, and participants responded only to red stimuli. Test trials consisted of 5 acquisition trials (5 blue) and 22 trials (11 blue and 11 red in random order) each in the discrimination and reverse-discrimination phases, with stimuli presented for 0.5 s at randomized interstimulus intervals of 1–2 s. Practice trials were completed before testing. Reaction time (RT), correct responses, correct inhibitions, Go errors (omissions), and No-go errors (commissions) were recorded. Responses occurring within 100 ms of stimulus onset were excluded. Task performance was evaluated using Go-task accuracy, No-go task accuracy, and correct response time (CRT), calculated as the mean RT for correct Go responses.

### Stroop test

Cognitive flexibility was assessed using a computerized Stroop test implemented with the Multi-Trigger System [22]. The task consisted of two congruent conditions (word reading (WR) and color naming (CN)) and one incongruent condition (color-word interference (CWI)). In WR, participants identified the meaning of color words presented in black font; in CN, they identified the color of colored circles; and in CWI, they identified the font color of incongruently colored words. Each condition consisted of 48 trials presented in random order with a stimulus duration of 1 s and an interstimulus interval of 1.5 s. Practice trials were conducted before testing. For each condition, RT, correct responses, incorrect responses, and omission errors were recorded. Responses occurring within 100 ms of stimulus onset were excluded. Task performance was evaluated based on accuracy and correct response time (CRT), calculated as the mean RT for correct responses. The difference in performance between the CWI and CN conditions was also calculated as an index of cognitive interference (Stroop effect).

### ANS activity

Heart rate variability was measured to assess ANS activity using a sphygmography device (TAS9 View; YKC Corp., Tokyo, Japan) [23]. All measurements were performed at the beginning of the visit with the participants being seated. Following the 150-s monitoring period, the device analyzed the standard deviation of normal-to-normal intervals (SDNN, reflecting overall ANS activity), low frequency (LF, representing sympathetic activity), high frequency (HF, representing parasympathetic activity) and the LF/HF ratio (an index of sympathovagal balance, with higher values indicating sympathetic dominance). The LF and HF values were transformed using natural logarithms and analyzed as LnLF and LnHF to approximate a normal distribution for statistical analysis.

### Assessment of emotion and behavior

The SDQ is a brief, validated screening tool for assessing emotional and behavioral problems in children and adolescents across the following domains: emotional symptoms, conduct problems, hyperactivity/inattention, peer problems, and prosocial behavior [24]. Each subscale consists of five items, with each item scored on a three-point scale (0 = not true, 1 = somewhat true, and 2 = certainly true). The total difficulty score was calculated as the sum of the four subscales (emotional symptoms, conduct problems, hyperactivity/inattention, and peer problems). Internalizing scores were derived from the sum of the emotional symptoms and peer problems subscales, whereas externalizing scores were derived from the sum of the conduct problems and hyperactivity/inattention subscales.

### Gut microbiota analysis

Fecal microbiota analysis was performed by TechnoSuruga Laboratory Co., Ltd. (Shizuoka, Japan). Gut microbiota composition was analyzed using terminal restriction fragment length polymorphism (T-RFLP) targeting the bacterial 16 S rRNA gene as previously described [25]. Briefly, fluorescently labeled terminal restriction fragments were generated by restriction enzyme digestion and analyzed by capillary electrophoresis to obtain community fingerprint profiles. Fragment polymorphisms were then grouped into operational taxonomic units (OTUs) representing major bacterial taxa (*Bifidobacterium*, *Coriobacteriaceae*, *Lactobacillales*, *Bacteroides*, *Prevotellaceae*, *Clostridium* cluster IV (*Ruminococcaceae*), subcluster XIVa (*Lachnospiraceae*), cluster IX (*Negativicutes*), *Akkermansia*, *Clostridium* cluster XI (*Peptostreptococcaceae*), cluster XVIII (*Erysipelotrichaceae*), and others).

### Procedure

The participants were tested individually in a quiet room at the study site, and all assessments were conducted during a single session lasting approximately 30 min. During this session, the participants underwent measurements of heart rate variability, digit span (forward and backward), Go/No-go task, and Stroop test, in this order. All assessments were conducted on non-school days (Saturdays or Sundays). At the baseline, and week 8 and 12 assessments, these tests were administered in the same order and within the same time window for each participant, with AM participants consistently assessed in the morning and PM participants consistently assessed in the afternoon (at the same time of the day for each participant).

### Safety

The safety analysis population was defined as all participants who received at least one sachet of the study food. All safety evaluations were conducted using a safety analysis population (Fig. 1). Safety was monitored throughout the study by reviewing diary records and interviewing participants and their parents. The parents were instructed to immediately inform the investigators of any adverse events, symptoms, or complaints during the study period. Parents also maintained a daily diary recording study food intake, use of medications or health supplements, subjective and objective symptoms, and any notable events related to daily life.

### Sample size

Due to the absence of sufficient prior data to perform a reliable power calculation, the sample size was determined with reference to previous studies with similar designs and outcome measures, including those by Hirayama et al. [26], together with practical feasibility considerations. A total of 60 participants was deemed adequate to provide preliminary evidence of efficacy and to inform the design of future confirmatory trials.

### Statistical analysis

The statistical analyses were performed using a predefined plan. The baseline characteristics and outcome measures were initially compared between the groups using parametric (Welch’s t-test) and nonparametric (Mann–Whitney U test, Wilcoxon signed-rank test, or chi-square test) statistical methods. Subsequently, for continuous cognitive outcomes (including digit span scores and reaction times), ANS measures, and SDQ scores, an analysis of covariance (ANCOVA) was used to compare post-intervention values between the groups, with baseline values included as covariates. Accuracy and error rates in the cognitive tasks, treated as binomial outcomes, were analyzed using generalized linear models with binomial distributions and logit link functions. Adjusted means were estimated for each group and between-group differences were reported as adjusted mean differences with 95% confidence intervals.

For the gut microbiota analysis, changes in each OTU at week 12 were analyzed using a negative binomial regression model adjusted for baseline values to account for the overdispersion commonly observed in the microbiome profiling data. In the alpha diversity analysis, Shannon and Simpson diversity indices were calculated, and between-group differences were assessed using the Wilcoxon rank-sum test. Beta diversity was assessed using Bray–Curtis dissimilarity and visualized using principal coordinate analysis (PCoA). Permutational multivariate analysis of variance (PERMANOVA) was used to evaluate the overall differences in community structure. Within-subject changes in beta diversity from baseline to 12 weeks were calculated based on Bray–Curtis distances to assess individual-level changes, and these changes were compared between groups using the Wilcoxon rank-sum test. A hierarchical testing strategy based on a closed testing procedure was applied to control for multiple comparisons across time points, in which the comparison at week 12 was conducted first and analyses at week 8 were subsequently performed only if a statistically significant group difference was observed at week 12. *p* < 0.05 was considered statistically significant. No missing data were observed; therefore, an imputation method was not used.

### Protocol availability

The study protocol is available at the UMIN Clinical Trials Registry (UMIN000058366, https://center6.umin.ac.jp/cgi-open-bin/icdr_e/ctr_view.cgi?recptno=R000066725) and from the corresponding author on reasonable request.

## Results

Of the 60 participants enrolled in the study, four (three in the postbiotic group and one in the placebo group) dropped out before the intervention because they were unable to attend the baseline outcome assessment. Additionally, one participant in the placebo group dropped out because of an inability to attend the 12-week outcome assessment. The remaining 55 participants completed the trial: 27 in the postbiotic group and 28 in the placebo group. According to the predefined criteria, one participant in the postbiotic group was excluded from the analysis because the consumption rate of the study food was below 80%, and two participants in the placebo group were excluded because of the intake of prohibited food (Fig. 1). The remaining participants showed a consumption rate of ≥ 90% (mean ±standard deviation (SD): 99.0 ± 1.8%), indicating high adherence to the intervention.

### Baseline characteristics of the participants and results of baseline outcomes

The clinical characteristics of the participants are presented in Table 2 and are categorized by the intervention group. In the postbiotic group (*N* = 26), 15 participants were third graders and 11 were fourth graders, whereas in the placebo group (*N* = 26), 14 participants were third graders and 12 were fourth graders. The postbiotic and placebo groups had 13 males and 13 females, and 12 males and 14 females, respectively. Statistical analyses did not show a significant difference in any baseline characteristics between the groups, including grade (*p* = 0.780), sex (*p* = 0.781), or other characteristics. The total scores on the ADHD-RS and SCQ were minimal, and no clinically relevant signs of ASD or ADHD were observed in the study population.

**Table 2:** Baseline clinical characteristics of participants in postbiotic and placebo group. Statistical analyses for grade and sex were performed using the chi-squared test. The Rohrer index is presented as mean ± standard deviation (SD) and was analyzed using Welch’s t-test. ADHD‑RS and SCQ scores are presented as median (interquartile range) and were compared between groups using Mann–Whitney U test

Variable		Postbiotic (*N* = 26)	Placebo (*N* = 26)	*p*-value
Grade	3rd grade	15	14	0.780
	4th grade	11	12	
Sex	Male	13	12	0.781
	Female	13	14	
Rohrer index	Male	123.2 ± 12.9	123.1 ± 9.6	0.984
	Female	126.9 ± 14.2	124.1 ± 11.2	0.591
ADHD-RS score	Inattention (0–27)	0.0 (0.0–1.8)	0.0 (0.0–2.0)	0.984
	Hyperactivity / Implusivity (0–27)	0.0 (0.0–0.0)	0.0 (0.0–0.0)	0.958
	Total (0–54)	0.0 (0.0–2.0)	0.0 (0.0–2.0)	1.000
SCQ score	(0–40)	1.0 (0.0–2.8)	0.0 (0.0–1.0)	0.397

Baseline outcomes for the primary endpoint are presented in Supplementary Tables S1A and S1B. The baseline clinical parameters of the primary endpoint assessment were generally comparable between the groups; however, statistically significant differences were observed in several measures. Because group allocation was based on screening scores obtained prior to the baseline assessment, minor imbalances in some baseline parameters were observed. Therefore, all subsequent analyses were adjusted for the baseline values, as detailed in Supplementary Tables S1A and S1B.

### Primary endpoints

**Fig. 2:**
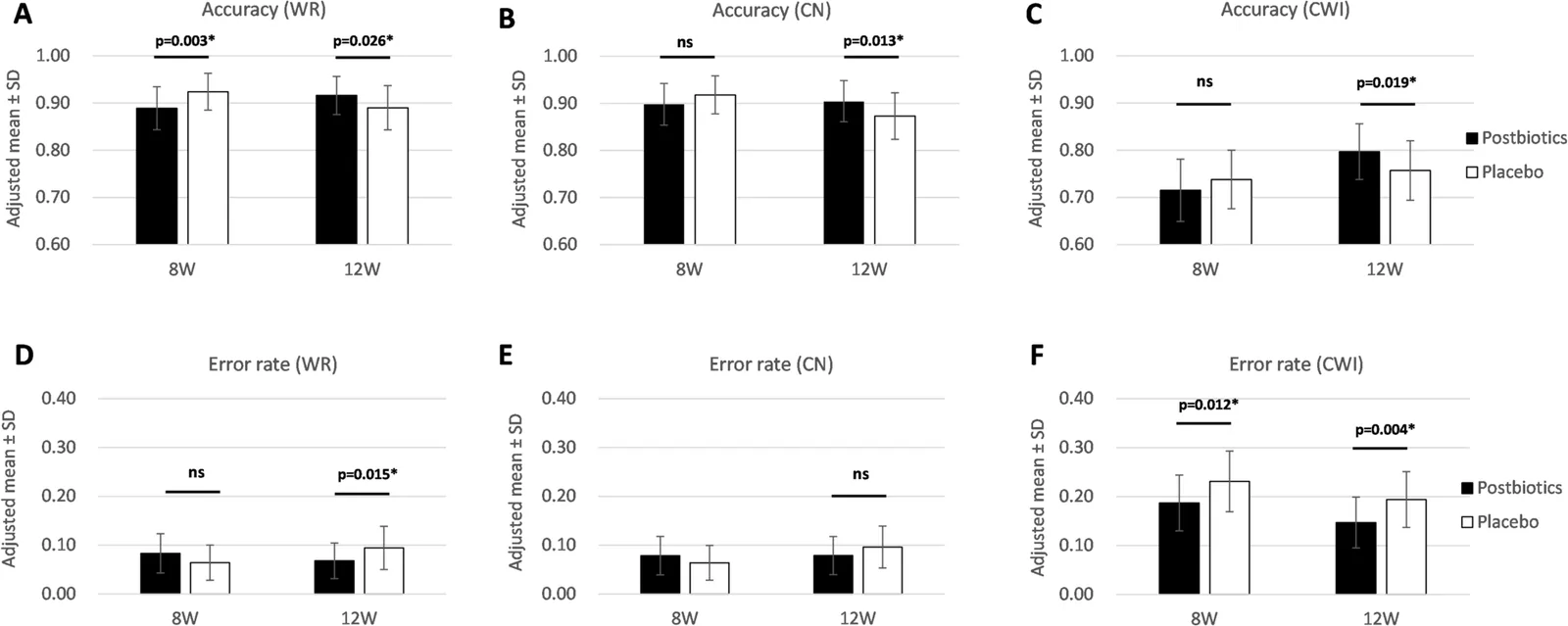
Stroop task performance, the primary cognitive outcome showing significant intervention effect. Baseline adjusted Stroop test accuracy and error rate in the congruent (WR, CN) and incongruent (CWI) conditions are demonstrated. Accuracy (**A**-**C**) and error rate (**D**-**F**) were adjusted for baseline scores, and p-values were calculated using a generalized linear model with a binomial distribution and logit link function, with baseline scores included as covariates. *p < 0.05; ns, not significant

For the primary endpoints, between-group comparisons at 12 weeks were performed using a closed testing procedure (Supplementary Table S1A). Among the cognitive assessments included in the primary endpoints, statistically significant intervention effects were observed primarily in the accuracy-related outcomes of the Stroop task. No significant differences (ANCOVA) were observed between the postbiotic and placebo groups in either the forward or backward digit span scores at 12 weeks. Similarly, the Go/No-go task showed no significant between-group differences in reaction time (ANCOVA) or accuracy (binomial generalized linear models). In contrast, participants who received the postbiotic intervention showed significant enhancement in the Stroop test accuracy (Fig. 2A-F), characterized by a higher correct response rate (accuracy: adjusted mean difference, 0.040 (95% CI 0.006–0.073, *p* = 0.019)) and a lower incorrect response rate (error rate: adjusted mean difference, -0.047 (95% CI -0.078– -0.015, *p* = 0.004)) in the incongruent condition at 12 weeks (Fig. 2C, F), noting that these metrics are not strictly complementary due to the presence of premature/invalid responses. In the congruent condition, the postbiotic intervention also significantly enhanced the accuracy of WR (accuracy: adjusted mean difference, 0.026 (95% CI 0.003–0.050, *p* = 0.026); error rate: adjusted mean difference, -0.026 (95% CI -0.048–-0.005, *p* = 0.015)) (Fig. 2A, D) and CN (accuracy: adjusted mean difference, 0.031 (95% CI 0.006–0.055, *p* = 0.013)) (Fig. 2B).

Following the closed-testing procedure, outcomes that showed significant between-group differences at 12 weeks were evaluated at 8 weeks using the same analytical approach (Supplementary Table S1B). In the incongruent condition, the postbiotic intervention significantly reduced the error rate at 8 weeks, consistent with the effect observed at 12 weeks (error rate: adjusted mean difference, -0.044 (95% CI -0.078– -0.010, *p* = 0.012)) (Fig. 2F). In contrast, in the congruent condition (WR), the accuracy was significantly higher in the placebo group, which was inconsistent with the results observed at 12 weeks (accuracy: adjusted mean difference, -0.035 (95% CI -0.058–-0.013, *p* = 0.003)). As the WR task primarily reflects automated processing rather than interference control, these inconsistent results across time points suggest that these differences are unlikely to represent stable changes in cognitive flexibility. Taken together, these results suggested that the most consistent changes were observed in the incongruent condition. Accordingly, the subsequent interpretations focused on this outcome.

Although significant between-group differences were observed in the Stroop test accuracy, accuracy-based interference, defined as the difference in accuracy between congruent (CN) and incongruent (CWI) conditions, did not differ significantly between the groups at 12 weeks (Supplementary Table S2). Regarding reaction time, no Stroop interference effect was observed during the assessment period and no significant between-group differences in reaction time interference were detected at 12 weeks (Supplementary Tables S1A, S2). These results indicate that the observed changes were limited to accuracy and did not extend to interference measures.

### Secondary endpoints

**Fig. 3:**
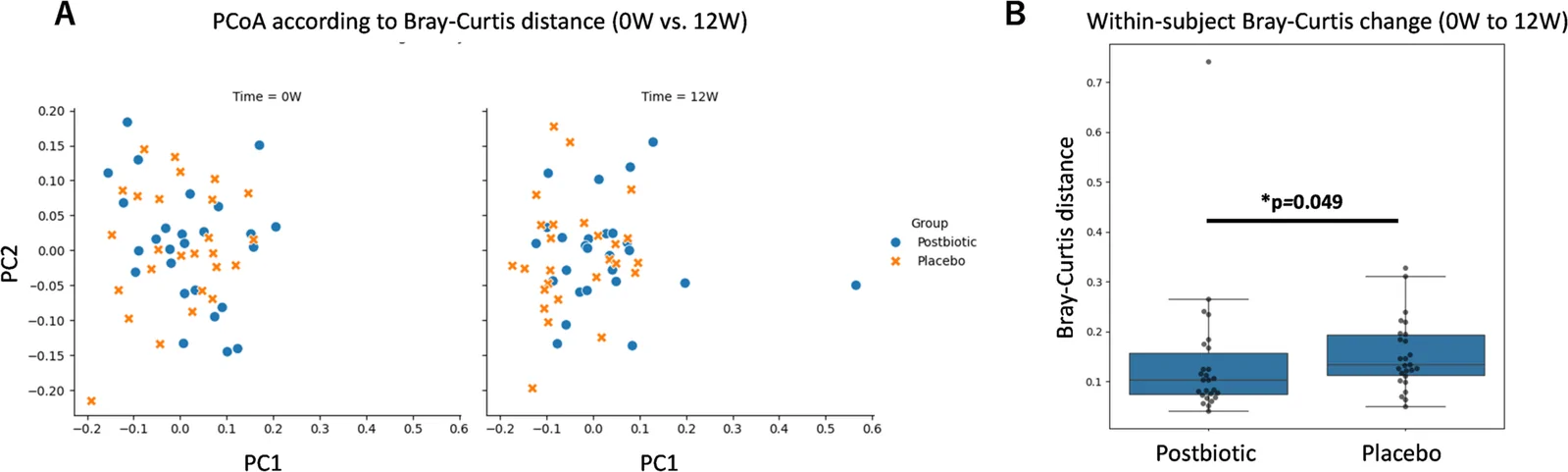
Changes in gut microbiota beta diversity in the postbiotic and placebo groups. **A** Principal coordinate analysis (PCoA) of gut microbiota based on Bray–Curtis dissimilarities. Separate panels showed baseline (week 0) and week 12 findings. No distinct group-level clustering was observed at either time point. **B** Within-subject Bray–Curtis distances between baseline and week 12 in the postbiotic and placebo groups. Boxplots show the distribution of individual-level microbiota changes. The p value was calculated using the Wilcoxon rank-sum test. *p < 0.05

Heart rate variability was evaluated using SDNN as a time-domain measure and LF and HF components as frequency-domain measures of ANS activity at baseline, week 8, and week 12. No significant between-group differences were observed at week 12 when analyzed using ANCOVA adjusted for baseline values (Supplementary Table S3). Similarly, only minimal changes were observed in the scores of each SDQ subscale, and no significant between-group differences were found at week 12 (Supplementary Table S4). Additionally, no significant between-group differences were observed in the gut microbiota analyses results after multiple testing corrections at week 12 ( Supplementary Table S5). Therefore, we shifted our focus from the individual taxa to the overall structure of the gut microbiota. We evaluated alpha and beta diversity changes between the groups to better capture community-level changes. In the alpha diversity analysis, no significant differences were observed in the Shannon or Simpson indices between the groups at baseline and week 12 (Supplementary Figure S1), indicating that the intervention did not significantly affect within-sample microbial diversity. Therefore, changes in beta diversity were assessed based on Bray–Curtis dissimilarity to further understand the overall differences in the gut microbiota composition between the groups. PCoA plots based on Bray–Curtis distance did not show distinct clustering by group or time point (baseline and 12 weeks, Fig. 3A), and PERMANOVA revealed no significant effects on the overall structure of the gut microbiota composition. However, individual-level changes in beta diversity from baseline to 12 weeks, quantified by the changes in Bray–Curtis distance, showed statistically significant differences between the groups (*p* = 0.049) (Fig. 3B). This change was greater in placebo group than that in the postbiotic group.

### Safety

During the intervention period, adverse events were reported by 10 and 16 participants in the postbiotic and placebo groups, respectively. However, no significant difference in the incidence of adverse events was observed between the groups. The most frequently reported events were mild respiratory symptoms including fever, cough, and sore throat (Supplementary Table S6). No serious adverse events were observed. According to the investigators’ assessment, all adverse events were deemed to be unrelated to the study foods, and no adverse events related to the intervention were observed.

## Discussion

Notably, our results demonstrated that intervention with KABP-042 and KABP-051 postbiotics enhanced the cognitive task performance demonstrated by the Stroop test accuracy in the incongruent condition, which was characterized by a higher correct response rate and a lower incorrect response rate at 12 weeks. In the incongruent condition, children had to temporarily inhibit the automatic response of reading the word and shift the response rule to naming the color, reflecting an increased cognitive demand, particularly in attention and cognitive control. This condition involves multiple cognitive processes, including inhibition of the automated response, selective attention to color information, and rule-switching under attentional control. Given these cognitive demands (sustaining attention, suppressing interference from irrelevant information, and maintaining goal-directed behavior), the present results suggest that postbiotic interventions may support task accuracy and attentional stability in children. This set of cognitive processes is collectively referred to as the executive function [27], which is essential for goal-directed behavior and learning in children. Thus, the present results indicate that the interventions with KABP-042 and KABP-051 postbiotics may selectively support performance on a cognitive task under conditions of increased cognitive demand in children. Executive function is closely linked to attention regulation, classroom behavior, and academic achievement in children [28]. Therefore, the observed enhancement in Stroop task accuracy may be relevant to cognitive processes that support successful learning and goal-directed behavior. Nevertheless, because academic performance was not assessed in the present study, further research is needed to determine the practical significance of these changes in real-world settings.

Although Stroop accuracy increased following the intervention, reaction time and the Stroop effect did not significantly change in this study. Accordingly, the observed changes in accuracy do not necessarily reflect changes in cognitive flexibility, which is more closely associated with reaction time measures and interference control. The absence of significant changes in reaction time and the Stroop effect may be due to several possible reasons. One possible explanation is that automatic word reading skills may not have been fully developed in the children in this study, which resulted in reduced interference during color naming. Schwanenflugel et al. [29] demonstrated that Stroop interference depends on reading automaticity, while Schadler and Thissen [30] reported that interference peaks at an intermediate stage of reading development. Even in third- and fourth-grade children, word reading still undergoes automatization; therefore, incomplete automatization may result in reduced or undetectable Stroop interference. Another possibility is that children may adopt a speed-oriented response strategy that focuses on color information while actively suppressing word processing. This speed-accuracy tradeoff is widely recognized in cognitive tasks [31], and several studies on children have reported that Stroop interference tends to be expressed in terms of accuracy rather than reaction time [32, 33]. Given that word reading is not yet fully automatized in children, this strategy may have eliminated lexical interference, leading to faster responses, even in incongruent conditions. Collectively, these results indicate that Stroop interference may be attenuated in reaction time but remains detectable in accuracy, and postbiotic intervention resulted only in enhanced accuracy, particularly in incongruent conditions.

Executive function consists of three core components, including inhibitory control, working memory, and cognitive flexibility [27, 28]. In the present study, although an effect on task accuracy related to cognitive flexibility was observed, no significant intervention effect was found in the digit span assessment. The forward task primarily reflects short-term memory capacity, whereas the backward task additionally requires the manipulation of information and is therefore considered a measure of working memory. We speculate that this may be attributable, at least in part, to the inter-individual variability typically observed in digit span performance, which may mask subtle intervention-related effects. In addition, we did not observe significant intervention-related effects on the Go/No-go task performance. A possible explanation for this result is the mismatch between task difficulty and participant characteristics, which may have limited sensitivity to change over time. Given that the participants were typically developing children, the baseline performance may have been close to the upper bound, resulting in a ceiling effect and leaving little room for enhancement. In addition, the duration and/or dose of the intervention may not have been sufficient to elicit measurable changes during this task. Therefore, future studies with more sensitive cognitive measures may help clarify intervention-related effects on working memory and inhibitory responses in children.

The results of the secondary outcome measures did not provide clear evidence of the intervention effects, which suggests that the effect of the intervention may be domain-specific, primarily affecting cognitive processes rather than psychophysiological or behavioral measures. ANS activity is characterized by substantial interindividual variability; therefore, more precise and sensitive assessment strategies may be required to fully elucidate intervention-related effects in this domain. In this study, emotional and behavioral functioning were assessed using the SDQ; however, the scores were uniformly low among the participants in the present study, who were typically developing children, suggesting the absence of notable emotional and behavioral difficulties. Given that children with high ADHD-RS-IV and SCQ scores were excluded during screening, this distribution was not unexpected and likely limited the sensitivity of the questionnaires in capturing potential intervention effects. Finally, the present study did not demonstrate significant changes in gut microbiota composition at the OTU level. The T-RFLP approach captures the overall structure of the gut microbial community; therefore, our analysis focused on global shifts in the microbial community structure rather than changes in individual OTUs. Consistent with this, no significant changes were observed in alpha diversity, suggesting that within-sample diversity remained stable throughout the intervention period. Consequently, we examined beta diversity, which captures inter-individual shifts in the microbial community composition, to assess whether the intervention induced different patterns of microbiota changes between the postbiotic and placebo groups. Interestingly, changes in the gut microbiota composition over the intervention period were smaller in the postbiotic group than in the placebo group. One possible explanation is that postbiotic supplementation may have attenuated natural temporal fluctuations in gut microbial community structure during the intervention period. Because the gut microbiota of school-aged children is continuously influenced by dietary habits and environmental factors, temporal fluctuations in microbial community composition can occur even in the absence of a specific intervention [11, 34]. Therefore, the greater beta diversity shift observed in the placebo group may reflect natural microbiota dynamics during the study periods, whereas postbiotic supplementation may have attenuated such fluctuations. Alternatively, the intervention may have exerted modest but distributed effects across multiple microbial groups, such that individual changes were too small to be detected by the analytical approach employed in this study, whereas their cumulative effect contributed to the maintenance of the community structure. However, these interpretations should be considered with caution. Importantly, gut microbiota composition was assessed using T-RFLP, which provides substantially lower taxonomic resolution than contemporary sequence-based approaches and generally does not permit reliable identification of microorganisms at the genus or species level. Consequently, subtle but biologically relevant microbial alterations potentially associated with the observed cognitive changes may have remained undetected. Therefore, the absent significant OTU-level differences should not be interpreted as definitive evidence that microbiota-mediated mechanisms were not involved in the observed effects. Although we did not identify a clear causal relationship between gut microbiota changes and cognitive task performance, the possibility that microbiota-related mechanisms contributed to the observed findings cannot be excluded. Further studies employing higher-resolution approaches, including 16 S rRNA gene amplicon sequencing or shotgun metagenomics, are required to more comprehensively characterize microbiota responses to postbiotic intervention and clarify potential microbiota-related pathways underlying the cognitive outcomes.

As described above, the present study demonstrated changes in the task assessing cognitive flexibility, whereas no comparable changes were observed in the measures of inhibitory control or working memory. One possible explanation is that cognitive flexibility may be more sensitive to intervention-related modulation during childhood than other executive function domains. This may partly explain why intervention-related effects were observed in the Stroop test but not in the digit span or Go/No-go tasks. Diamond [28] described that cognitive flexibility represents a higher-order executive function that builds upon more fundamental components such as inhibitory control and working memory. Buttelmann and Karbach [35] also consistently described the plasticity of cognitive flexibility, reporting that it exhibits plasticity during early and middle childhood and can be modulated by targeted interventions. From this perspective, the observed changes in Stroop task performance, along with the absence of clear changes in inhibitory control and working memory, may reflect a domain-specific sensitivity to the intervention effect during this developmental period. However, this interpretation remains speculative and requires confirmation in future studies. Although the precise mechanisms underlying the observed cognitive changes remain unclear, several gut–brain communication pathways have been proposed. Previous studies suggest that microbiome-targeted interventions may influence neurotrophic signaling pathways including BDNF, modulate gut-derived hormones such as GLP-1, and affect neural communication through the vagus nerve [18, 36, 37]. These pathways may contribute to the regulation of attention and cognitive performance. However, additional studies are required to elucidate the extent to which BDNF-mediated neuroplasticity contributes to the selective changes in Stroop task performance observed in this study. Furthermore, as BDNF, GLP-1, and other mechanistic biomarkers were not measured in the present study, the involvement of these pathways remains unclear and should be examined in future studies.

To date, evidence on the effects of probiotic or postbiotic interventions on brain function in children is limited, particularly in studies involving healthy populations, with most reported effects observed in children with conditions such as ASD or ADHD [38–40]. From this perspective, the present study provides important preliminary evidence that such interventions may influence performance on specific cognitive tasks in typically developing children. In addition, no adverse events related to the postbiotic intervention were observed during the 12-week intake period, supporting the favorable safety and tolerability profile in this study.

### Limitations

This study has some limitations that should be considered when interpreting the results. First, this study was exploratory in nature with a relatively small sample size (*N* = 60). Although statistically significant effects were observed for some outcomes, further clinical research with a larger sample size is required to obtain more generalizable evidence of efficacy. In addition, efficacy analyses were conducted using the per-protocol population rather than an ITT approach. Although participant compliance was high and the dropout rate was limited, the absence of an ITT analysis may have introduced potential bias in the estimation of treatment effects. Further studies should employ both ITT and per-protocol analyses to confirm the robustness of the findings. Second, the intervention period was limited to 12 weeks; therefore, the effects of long-term interventions on brain function could not be evaluated. While the present study detected changes in Stroop task performance, its effects on other domains of cognitive function such as inhibitory control and working memory remain to be investigated in future studies. Third, the participants were typically developing children without ASD or ADHD. Accordingly, the potential effects of the intervention may have been difficult to determine. Therefore, whether similar or greater benefits are observed in children with mild neurodevelopmental characteristics remains an important topic for future studies. Fourth, although the intake of prohibited supplements and functional foods was restricted during the intervention period, overall dietary intake was not systematically assessed. Therefore, we cannot exclude the possibility that differences in habitual dietary patterns may have influenced cognitive outcomes, autonomic nervous system activity, or gut microbiota composition. Fifth, we were unable to detect differences in the digit span or Go/No-go tasks. Consequently, further refinement of the study design and development of more sensitive assessment tools may be necessary to adequately detect intervention-related changes in these domains. Sixth, regarding the Stroop test outcomes, although a significant effect was observed on accuracy measures, no significant effects were detected on Stroop interference or reaction time. These findings indicate that the current assessment may not have been sufficient to comprehensively capture the changes in all aspects of cognitive flexibility. Therefore, the results should not be interpreted as evidence of an overall enhancement in cognitive flexibility. The present findings are limited to enhanced accuracy-related Stroop task performance under the conditions employed in this study. Furthermore, because statistically significant findings were confined to a limited number of outcomes among multiple assessments, the possibility of a chance finding cannot be completely excluded. Further studies using refined assessment paradigms and adequately powered samples are required to better understand these effects. Finally, in addition to the limitation related to cognitive assessment, the interpretation of the microbiota findings should also be considered with caution. Although T-RFLP provides an overview of community structure, its taxonomic resolution is substantially lower than that of contemporary sequencing-based approaches such as 16 S rRNA gene amplicon sequencing and shotgun metagenomics. Consequently, subtle but biologically relevant microbial alterations that may have contributed to the observed cognitive effects could have remained undetected. Therefore, the absence of significant differences in OTU-level composition should not be interpreted as definitive evidence that microbiota-mediated mechanisms were not involved in the observed effects. Future studies employing higher-resolution microbiome analyses will be required to more comprehensively characterize microbiota responses to postbiotic intervention.

## Conclusions

This exploratory, randomized, placebo-controlled trial demonstrated that a 12-week postbiotic intervention with heat-killed *B. longum* KABP-042 and *L. plantarum* KABP-051 selectively enhanced accuracy-related performance on the Stroop task, particularly under incongruent conditions, in typically developing school-aged children. No significant effects were observed on working memory, inhibitory control, ANS activity, emotional and behavioral outcomes, and overall gut microbiota composition at the OTU level. These findings provide preliminary clinical evidence that specific postbiotic strains may influence selected aspects of cognitive performance in children. However, given that significant effects were observed in a limited set of outcomes, the results should be interpreted with caution. Further investigation in larger, adequately powered, and longer-term studies is needed to confirm these findings and to clarify the underlying gut-brain mechanisms of postbiotic supplementation.

## Supplementary Information

Supplementary Material 1: Supplementary Fig. S1. Comparison of alpha diversity indices between postbiotic and placebo groups.

Supplementary Material 2: Supplementary Table S1A. Primary endpoint outcomes and between-group comparisons after 12 weeks of intervention.

Supplementary Material 3: Supplementary Table S1B. Primary endpoint outcomes and between-group comparisons after 8 weeks of intervention.

Supplementary Material 4: Supplementary Table S2. Baseline-adjusted Stroop interference at 12 weeks.

Supplementary Material 5: Supplementary Table S3. Changes in ANS measures in postbiotic and placebo group.

Supplementary Material 6: Supplementary Table S4. Changes in SDQ scores in postbiotic and placebo group.

Supplementary Material 7: Supplementary Table S5. Effects of postbiotic intervention on gut microbiota composition at baseline and week 12 and between-group differences estimated by negative binomial regression.

Supplementary Material 8: Supplementary Table S6. Adverse events including subjective and objective symptoms analyzed on a per-subject basis

## Data Availability

The datasets generated and/or analyzed during the current study are not publicly available because of privacy and ethical restrictions involving human participants but are available from the corresponding author upon reasonable request.

## Abbreviations

ADHD: attention-deficit/hyperactivity disorder
ADHD-RS: Attention-Deficit/Hyperactivity Disorder Rating Scale
ANCOVA: analysis of covariance
ANS: autonomic nervous system
ASD: autism spectrum disorder
BDNF: brain-derived neurotrophic factor
CN: color-naming
CRT: correct response time
CWI: color-word interference
GLP-1: glucagon‑like peptide‑1
HF: high frequency
ITT: intention-to-treat
LF: low frequency
OTU: operational taxonomic unit
PCoA: principal coordinates analysis
PERMANOVA: permutational multivariate analysis of variance
RT: reaction time
SCQ: Social Communication Questionnaire
SD: standard deviation
SDNN: standard deviation of normal-to-normal intervals
SDQ: Strengths and Difficulties Questionnaire
T-RFLP: terminal restriction fragment length polymorphism
WISC: Wechsler Intelligence Scale for Children
WR: word-reading

## References

[1] Cryan JF, Dinan TG. Mind-altering microorganisms: the impact of the gut microbiota on brain and behavior. Nat Rev Neurosci. 2012;13:701–12. 10.1038/nrn3346. 22968153

[2] Sarkar A, Lehto SM, Harty S, Dinan TG, Cryan JF, Burnet PWJ. Psychobiotics and the manipulation of brain function. Trends Cogn Sci. 2016;20(9):611–23. 10.1016/j.tics.2016.01.002. PMC5102282 27793434

[3] Asaoka D, Xiao J, Takeda T, Yanagisawa N, Yamazaki T, Matsubara Y, et al. Effect of probiotic Bifidobacterium breve MCC1274 in improving cognitive function and preventing brain atrophy in older patients with suspected mild cognitive impairment: results of a 24-week randomized, double-blind, placebo-controlled trial. J Alzheimers Dis. 2022;88(1):75–95. 10.3233/JAD-220148. PMC9277669 35570493

[4] Azuma N, Mawatari T, Saito Y, Tsukamoto M, Sampei M, Iwama Y. Effect of continuous ingestion of Bifidobacterium animalis subsp. lactis GCL2505 and dietary fiber on improvement in cognitive function: a randomized, double-blind, placebo-controlled trial. Nutrients. 2023;15(19):4175. 10.3390/nu15194175. PMC10574581 37836458

[5] Xiao J, Katsumata N, Bernier F, Ohno K, Yamauchi Y, Odamaki T, et al. Probiotic Bifidobacterium breve A1 improves cognitive function in older adults with suspected mild cognitive impairment: a randomized, double-blind, placebo-controlled trial. J Alzheimers Dis. 2020;77(1):139–47. 10.3233/JAD-200488. PMC7592675 32623402

[6] Messaoudi M, Lalonde R, Violle N, Javelot H, Desor D, Nejdi A, et al. Assessment of psychotropic-like properties of a probiotic formulation (*Lactobacillus helveticus* R0052 and *Bifidobacterium longum* R0175) in rats and human subjects. Br J Nutr. 2011;105(5):755–64. 10.1017/S0007114510004319. 20974015

[7] Schaub AC, Schneider E, Vazquez-Castellanos JF, Schweinfurth N, Kettelhack C, Doll JPK, et al. Clinical, gut microbial and neural effects of a probiotic add-on therapy in depressed patients: a randomized controlled trial. Transl Psychiatry. 2022;12:227. 10.1038/s41398-022-01977-z. PMC9163095 35654766

[8] Mutoh N, Kakiuchi I, Hiraku A, Iwabuchi N, Kiyosawa K, Igarashi K, et al. Heat-killed *Lactobacillus helveticus* MCC1848 improves mood states: a randomised, double-blind, placebo-controlled study. Benef Microbes. 2023;14(2):109–18. 10.3920/BM2022.0048. 37026368

[9] Watanabe T, Kurosaka S, Namatame Y, Kawahara T. Effect of heat-killed *Lactiplantibacillus plantarum* SNK12 on sleep quality and stress-related neuroendocrine and inflammatory biomarkers in adults: a randomized, double-blind, placebo-controlled, parallel-group trial. Life. 2025;16(1):26. 10.3390/life16010026. PMC12843449 41598182

[10] Kavita H, Om, Chand U, Kushawaha PK. Postbiotics: an alternative and innovative intervention for the therapy of inflammatory bowel disease. Microbiol Res. 2024;279:127550. 10.1016/j.micres.2023.127550. 38016379

[11] Cryan JF, O’Riordan KJ, Cowan CSM, et al. The microbiota–gut–brain axis. Physiol Rev. 2019;99:1877–2013. 10.1152/physrev.00018.2018. 31460832

[12] Maqsood R, Stone TW. The gut–brain axis, BDNF, NMDA and CNS disorders. Neurochem Res. 2016;41:2819–35. 10.1007/s11064-016-2039-1. 27553784

[13] Salami M, Soheili M. The microbiota–gut–hippocampus axis. Front Neurosci. 2022;16:1065995. 10.3389/fnins.2022.1065995. PMC9817109 36620458

[14] Fukuchi M, Arai N, Honda S, Tominaga Y. Heat-killed *Bifidobacterium longum* KABP-042 and *Lactiplantibacillus plantarum* KABP-051 prevent scopolamine-induced BDNF reduction and memory impairment in mice. Cell Biochem Biophys. 2026. 10.1007/s12013-026-02057-5. PMC13585778 41831189

[15] Ohta K, Suzuki S, Warita K, Kaji T, Kusaka T, Miki T. Prolonged maternal separation attenuates BDNF-ERK signaling correlated with spine formation in the hippocampus during early brain development. J Neurochem. 2017;141(2):179–94. 10.1111/jnc.13977. 28178750

[16] Ohta K, Ujihara H, Kumei H, Suzuki S, Otabi H, Warita K, et al. Effects of attenuated TrkB signaling on the medial prefrontal cortex during early brain development: a comparative study using the maternal separation model. Behav Brain Res. 2025;495:115790. 10.1016/j.bbr.2025.115790. 40889559

[17] Rutter M, Bailey A, Lord C. The Social Communication Questionnaire. Los Angeles: Western Psychological Services; 2003.

[18] DuPaul GJ, Power TJ, Anastopoulos AD, Reid R. ADHD Rating Scale-IV: Checklists, norms, and clinical interpretation. New York: Guilford Press; 1998.

[19] Wechsler D. Wechsler Intelligence Scale for Children–Fifth Edition (WISC-V): Technical and interpretive manual. San Antonio (TX): Pearson; 2014.

[20] Watanabe N, Kamijo M, Ashida K, Sasamori F, Okuhara M, Maruo SJ, et al. Early health education and cognitive inhibitory control in children: a 20-year pilot study using the go/no-go task in Japan. Front Public Health. 2025;13:1703017. 10.3389/fpubh.2025.1703017. PMC12675476 41358209

[21] Terasawa K, Tabuchi H, Yanagisawa H, Yanagisawa A, Shinohara K, Terasawa S, et al. Comparative survey of go/no-go results to identify the inhibitory control ability change of Japanese children. Biopsychosoc Med. 2014;8:14. 10.1186/1751-0759-8-14. PMC4109780 25061475

[22] Scarpina F, Tagini S. The Stroop Color and Word Test. Front Psychol. 2017;8:557. 10.3389/fpsyg.2017.00557. PMC5388755 28446889

[23] Yoshida Y, Takei H, Ukisu M, Takagi K, Tanito M. Association of heart rate variability and acceleration plethysmography with systemic comorbidity burden in patients with glaucoma. Biomedicines. 2025;13(9):2155. 10.3390/biomedicines13092155. PMC12467616 41007717

[24] Goodman R. The Strengths and Difficulties Questionnaire: a research note. J Child Psychol Psychiatry. 1997;38(5):581–6. 10.1111/j.1469-7610.1997.tb01545.x. 9255702

[25] Nagashima K, Hisada T, Mochizuki J. Analysis of intestinal microbiota using T-RFLP. J Intest Microbiol. 2014;28(4):155–64. 10.11209/jim.28.155.

[26] Hirayama S, Terasawa K, Rabeler R, Hirayama T, Inoue T, Tatsumi Y, et al. The effect of phosphatidylserine administration on memory and symptoms of attention-deficit hyperactivity disorder: a randomised, double–blind, placebo–controlled clinical trial. J Hum Nutr Diet. 2014;27(Suppl 2):284–91. 10.1111/jhn.12090. 23495677

[27] Miyake A, Friedman NP, Emerson MJ, Witzki AH, Howerter A, Wager TD. The unity and diversity of executive functions and their contributions to complex frontal lobe tasks. Cogn Psychol. 2000;41(1):49–100. 10.1006/cogp.1999.0734. 10945922

[28] Diamond A. Executive functions. Annu Rev Psychol. 2013;64:135–68. 10.1146/annurev-psych-113011-143750. PMC4084861 23020641

[29] Schwanenflugel PJ, Morris RD, Kuhn MR, Strauss GP, Sieczko JM. The influence of reading unit size on the development of Stroop interference in early word decoding. Read Writ. 2008;21(3):177–203. 10.1007/s11145-007-9061-2. PMC2772162 19890455

[30] Schadler M, Thissen DM. The development of automatic word recognition and reading skill. Mem Cognit. 1981;9(2):132–41. 10.3758/BF03202327. 7242326

[31] Heitz RP. The speed–accuracy tradeoff: history, physiology, methodology, and behavior. Front Neurosci. 2014;8:150. 10.3389/fnins.2014.00150. PMC4052662 24966810

[32] Prevor MB, Diamond A. Color–object interference in young children: a Stroop effect in children 3½–6½ years old. Cogn Dev. 2005;20(2):256–78. 10.1016/j.cogdev.2005.04.001. PMC2134842 18079980

[33] Wright BC. What Stroop tasks can tell us about selective attention from childhood to adulthood. Br J Psychol. 2017;108(3):583–607. 10.1111/bjop.12230. PMC5516206 27786354

[34] Bankole T, Li Y. The early-life gut microbiome in common pediatric diseases: roles and therapeutic implications. Front Nutr. 2025;12:1597206. 10.3389/fnut.2025.1597206. PMC12158737 40510495

[35] Buttelmann F, Karbach J. Development and plasticity of cognitive flexibility in early and middle childhood. Front Psychol. 2017;8:876. 10.3389/fpsyg.2017.00876. PMC5476931 28676784

[36] Morais LH, Schreiber HL 4th, Mazmanian SK. The gut microbiota-brain axis in behaviour and brain disorders. Nat Rev Microbiol. 2021;19:241–55. 10.1038/s41579-020-00460-0. 33093662

[37] Joung JY, Choi HS, Oh NS. Emerging roles of postbiotics in gut-brain-microbiome axis modulation and neurobiological pathways of chronic stress-related brain dysfunction. J Microbiol Biotechnol. 2026;36:e2603010. 10.4014/jmb.2603.03010. PMC13146489 42049694

[38] Elhossiny RM, Elshahawy HH, Mohamed HM, Abdelmageed RI. Assessment of probiotic strain *Lactobacillus acidophilus* LB supplementation as adjunctive management of attention-deficit hyperactivity disorder in children and adolescents: a randomized controlled clinical trial. BMC Psychiatry. 2023;23(1):823. 10.1186/s12888-023-05324-4. PMC10636814 37946220

[39] Kumperscak HG, Gricar A, Ülen I, Micetic-Turk D. A pilot randomized control trial with the probiotic strain *Lactobacillus rhamnosus* GG (LGG) in ADHD: children and adolescents report better health-related quality of life. Front Psychiatry. 2020;11:181. 10.3389/fpsyt.2020.00181. PMC7092625 32256407

[40] Liu YW, Liong MT, Chung YCE, Huang HY, Peng WS, Cheng YF, et al. Effects of *Lactobacillus plantarum* PS128 on children with autism spectrum disorder in Taiwan: a randomized, double-blind, placebo-controlled trial. Nutrients. 2019;11(4):820. 10.3390/nu11040820. PMC6521002 30979038

